# Complications and Comorbidities in Hispanic Patients Who Develop Traumatic and Non-traumatic Acute Compartment Syndrome

**DOI:** 10.7759/cureus.12792

**Published:** 2021-01-19

**Authors:** Jorge Alejandro Bernal, Annelyn Torres-Reveron, Jose Andres Gonzalez

**Affiliations:** 1 Internal Medicine Residency Program, University of Texas Rio Grande Valley – DHR Health, Edinburg, USA; 2 Research, DHR Health Institute for Research and Development, Edinburg, USA; 3 Surgery, DHR Health Surgery Institute, McAllen, USA

**Keywords:** statins, anticoagulants, hypertension, comorbidities, fractures, extremities, risk factors, general trauma surgery

## Abstract

Acute compartment syndrome (ACS) is a medical emergency that remains under-recognized and understudied. This study aimed to identify risk factors for the traumatic and non-traumatic presentation of ACS within a majority Hispanic population. A four-year retrospective analysis of medical records in a single institution revealed 26 with traumatic and 21 non-traumatic patients presenting with ACS. Traumatic ACS occurred in younger males following fractures, as previously described in the field. After controlling for age differences, non-traumatic ACS occurred in older patients with multiple comorbidities, increased use of statins, and anticoagulants as compared to the traumatic ACS group. A large proportion (80%) of the non-traumatic ACS group also presented with hypertension. Patients taking anticoagulants and statins should be carefully monitored for ACS development after non-traumatic qualifying events and advanced age. Further studies should identify how statins interact with the patients' racial/ethnic profile and the incidence of comorbidities to promote earlier identification and reduce morbidities.

## Introduction

It is estimated that acute compartment syndrome (ACS) affects approximately 1%-7% of the world population annually [1,2]. This condition is classified as increased intercompartmental pressure due to ischemia and inflammation following an injury or reperfusion of the limb tissue. Although it is considered uncommon throughout other medical specialties, it is most commonly seen during orthopedic and trauma surgery [2]. Surgeons need to identify this emergent condition quickly to avoid morbidity and mortality in ACS.

The diagnosis of ACS remains challenging despite well-established signs and symptoms for its presentation [2]. Comatose or major trauma patients are challenging to diagnose because the classic presentation may not be straightforward [1]. Recognizing the risk factors is essential for a timely diagnosis and an early fasciotomy intervention. ACS risk factors include male patients above age 35, recent bone fractures (especially tibia fractures), soft tissue injuries, muscle ischemia, and even burns [3]. ACS can lead to muscle necrosis within three hours of muscle injury. The incidence of muscle necrosis is estimated to be 46% [4]. Muscle necrosis can then lead to other limb complications, which may ultimately include sepsis and amputations.

There is currently a limited amount of data on chronic conditions that may be risk factors for ACS and even less data on Hispanics' chronic risk factors. This gap in knowledge for a Hispanic population with high rates of chronic conditions may be inadvertently aggravating patients' prognosis with ACS. In the lower Rio Grande Valley (RGV), a region in Texas's southernmost tip where more than 90% of the population is of Hispanic/Latino heritage [5], the morbidity of ACS and the incidence has not been established. Also, the RGV has high rates of many chronic conditions and diseases such as coronary artery disease, obesity, and diabetes, many of which remain among the highest in the United States [6-9]. In this study, we analyzed patients with the diagnosis and clinical presentation of ACS, focusing on comparing traumatic and non-traumatic presentations. Our goal was not to find the incidence of ACS in our region but to identify the risk factors and comorbidities in patients for cases that involve traumatic and non-traumatic events. Based on a large number of comorbidities in our patient population, we hypothesize that ACS will present in patients with similar comorbidities, regardless of the triggering event. Identifying the clinical characteristics between traumatic and non-traumatic ACS events can inform the medical community about risk factors, especially for the Hispanic population and patients with multiple comorbidities.

## Materials and methods

A retrospective review of electronic medical records information from January 1, 2015, to December 31, 2019, was conducted within a Level 2 Trauma hospital in the United States' southernmost region. The study was approved by the Institutional Review Board and conformed to the Declaration of Helsinki and the U.S. Federal Policy for the Protection of Humans Subjects. All data collected were from documentation about the standard of care. A waiver of informed consent based on the 45 CFR 46.116 was requested and approved by the Institutional Review Board. The proposed research involves no more than minimal risk to the patients and could not be carried out practicably without this waiver.

The definition of traumatic and non-traumatic compartment syndrome was based on T79 and M79 International Coding Diagnosis (ICD 10) codes, with their respective modifiers for the lower and upper extremities. Since compartment syndrome might not always appropriately coded in the medical records, we also identified patients who underwent a fasciotomy using the following current procedural terminology codes: 27600, 27601, 27602, 27892, 27893, 27894. Compartment syndrome was identified based on documentation of the following criteria: pain out of proportion, pain in response to stretching the affected limb, paresthesia, tension, pallor, weakness, or paralysis [10,11]. Since limb amputation is a possible outcome of compartment syndrome, we retrospectively evaluated limb amputation cases to determine if the cause was a consequence of compartment syndrome. Patients were identified from all documented admissions to the main hospital and all affiliated clinics regardless if the patient presented via emergency room or regular office visits. One of the investigators assessed subject eligibility, and an independent trauma data analyst corroborated the inclusion criteria validity. Male and female patients were included in the study. Patients with compartment syndrome presentation in anatomical areas other than the upper or lower extremities were not accounted for in this study. In addition, patients presenting with osteomyelitis and gangrene were also excluded since the clinical and physiological presentation often follows a chronic progression. Patients younger than 18 years were excluded as per study design and IRB approval.

To extract the data, we first identified the patients who met the criteria for age, diagnosis, and procedure codes from the medical records at the hospital via a report from our site business intelligence department. Second, we documented the condition(s) for which the patient was seeking treatment: if the ACS resulted from a traumatic event, collect the trauma mechanism as reported in the medical chart; if there was no trauma involved, collect the clinical event that occurred before presentation. Third, we collected from the medical record the time and type of initial procedure (if any), neurovascular injury presence, chronic health conditions, length of stay, current medications, and prior traumas or surgical interventions in the same anatomical region of the identified compartment syndrome. Fourth, the collected demographic variables included the age at the time of the event, sex, race/ethnicity, and body mass index (BMI). All information were collected in a Microsoft Excel sheet using an alphanumeric code by a patient. No paper information was collected or retained for the study. 

Descriptive statistics were used for the entire study population. Frequencies and column percentages were used to summarize categorical variables. Continuous variables were tested for normality using the Shapiro-Wilk goodness-of-fit test. Non-normally distributed variables were compared using the Wilcoxon signed-rank test, and normally distributed variables were compared using the Student's t-test for independent samples. Chi-square or Fisher's exact tests were used for categorical variables. Logistic regression analyses were used to compare the effects of comorbidities and medications used in traumatic vs. non-traumatic ACS controlling for the patients' age. The statistical analyses were two-sided and conducted using JMP 15.0 (SAS Institute, Inc, Cary, NC, USA). The significance was set at p <0.05.

## Results

Participants and demographic characteristics

An initial sample of 286 patients was identified for review. Amputations accounted for 179 records, fasciotomies were an additional 52 records, and 55 records had an explicit diagnosis of compartment syndrome in the upper or lower extremities. Thirty-six of the records from patients in the fasciotomy procedure report were also in the compartment syndrome report. Exclusion criteria were applied to eliminate patients under 18 years (seven records) and osteomyelitis (42 records). Most of the remaining records were eliminated due to amputation resulting from gangrenous processes with no evidence of compartment syndrome, fasciotomies procedures for other clinical presentations, and coding errors (compartment syndrome in areas other than the limbs). Twenty-one non-traumatic and 26 traumatic records of patients with compartment syndrome were included in the study. Since patients were initially identified by procedures frequently used after ACS and explicit coding of the condition in the record, the true prevalence of ACS in our population cannot be calculated due to the study's inherent design. 

Table 1 describes the demographic characteristics of the cohort. Patients with traumatic ACS were on average 16 years younger than the patients with non-traumatic ACS, and this difference was significant (t = -3.04, d.f. = 41.12, p < 0.01). Traumatic ACS patients were predominantly male compared to non-traumatic ACS patients (X2 = 4.38, d.f. = 1, p < 0.05). Eighty percent of the cohort self-reported Hispanic/Latino ethnicity, which is usual for our geographical area. The BMIs of both traumatic and non-traumatic ACS were very similar, and both groups were considered obese. The traumatic group patients had a length of hospital stay of 3.85 days more than the non-traumatic ACS group, but this difference was not significant. The frequency of ACS was three times higher in the lower extremities than in the upper extremities in both groups. 

**Table 1:** Demographic variables BMI, body mass index; LOS, length of stay; CS, compartment syndrome

Variable	Non-traumatic (NT)	Traumatic (T)	p-Value, NT vs. T
Number of patients	21	26	
Age, mean (SD)	63.43 (18.78)	47.42 (17.17)	0.004
Sex, males, n (%)	12 (57.14)	22 (84.61)	0.039
Ethnicity, Hispanic (%)	17 (80.95)	21 (80.77)	0.647
BMI, mean (SD)	31.99 (6.90)	30.81 (8.55)	0.603
LOS, mean (SD)	9.38 (7.43)	13.23 (10.72)	0.154
CS, lower extremity	18 (85.71)	19 (73.08)	
CS, upper extremity	3 (14.29)	7 (26.92)	

Main outcomes

The clinical factors that may influence ACS development were cataloged in both groups and presented in Table 2. Neurovascular injury showed a trend toward significance with increased frequency in the non-traumatic group (X2 = 1.66, d.f. = 1, p = 0.06). The odds of neurovascular injury in the non-traumatic ACS were 3.83 (95% CI: 0.85-17.30) compared to the traumatic ACS. No statistical differences were obtained for the type of procedures performed between traumatic and non-traumatic groups (X2 = 1.27, d.f. = 3, p > 0.05). Since fasciotomies are the most frequent procedure performed after identifying ACS, we compared the odds ratio for fasciotomies in the non-traumatic group at 0.66 (95% CI: 0.04-11.56); fasciotomies were slightly less frequent in the non-traumatic group. Qualitative comparison of the clinical events before ACS presentation was different for each group, with vascular complications and ischemia frequently observed in the non-traumatic group and fractures in the traumatic group.

**Table 2:** Clinical characteristics of the cohort

Variables, n (%)	Non-traumatic (NT)	Traumatic (T)	Odds ratio (95% CI) for NT
Neurovascular injury	7 (33.33)	3 (11.54)	3.83 (0.85-17.30)
Procedures			
Fasciotomy	14 (66.67)	21 (80.77)	0.66 (0.04-11.56)
Amputation	1 (4.76)	1 (3.85)	
No procedure	5 (23.81)	1 (3.85)	
Other	1 (4.76)	3 (11.53)	
Cause/history			
Surgical complication	3 (14.28)	0	
Vascular complications	7 (33.33)	0	
Ischemia	4 (19.04)	0	
Exertional/muscle injection	2 (9.52)	1 (3.84)	
Fall-no fracture	0	7 (26.92)	
Fracture	0	11 (42.31)	
Penetrating injury	0	2 (7.69)	
Other/unknown	5 (23.80)	5 (19.23)	

The glucose and creatinine values before any intervention and post-intervention were documented and extracted from the charts for those patients for which it was documented (Table 3). The only significant difference between traumatic and non-traumatic groups was observed for post-procedure glucose. The non-traumatic group had a difference of 40 points higher than the traumatic ACS group (t = -2.91, d.f. = 17.51, p < 0.05). It is worth noting that glucose and creatinine changes followed opposite patterns in the cohort (see percent difference in Table 3). 

**Table 3:** Glucose and creatinine values for a sub-set of patients reported in the electronic medical record

Variables, mean ± SD, n	Non-traumatic (NT)	Traumatic (T)	p-Value, NT vs. T
Glucose, pre-procedure	143.81 ± 53.00, 11	172.88 ± 119.80, 18	0.379
Glucose, post-procedure	172.30 ± 45.00, 13	132.00 ± 24.80, 17	0.009
% change pre to post	19.81	-23.64	
Creatinine pre-procedure	3.14 ± 3.41, 11	1.20 ± 1.45, 22	0.095
Creatinine post-procedure	2.77 ± 2.91, 9	1.42 ± 1.91, 17	0.232
% change pre to post	-11.78	18.33	

**Figure 1:**
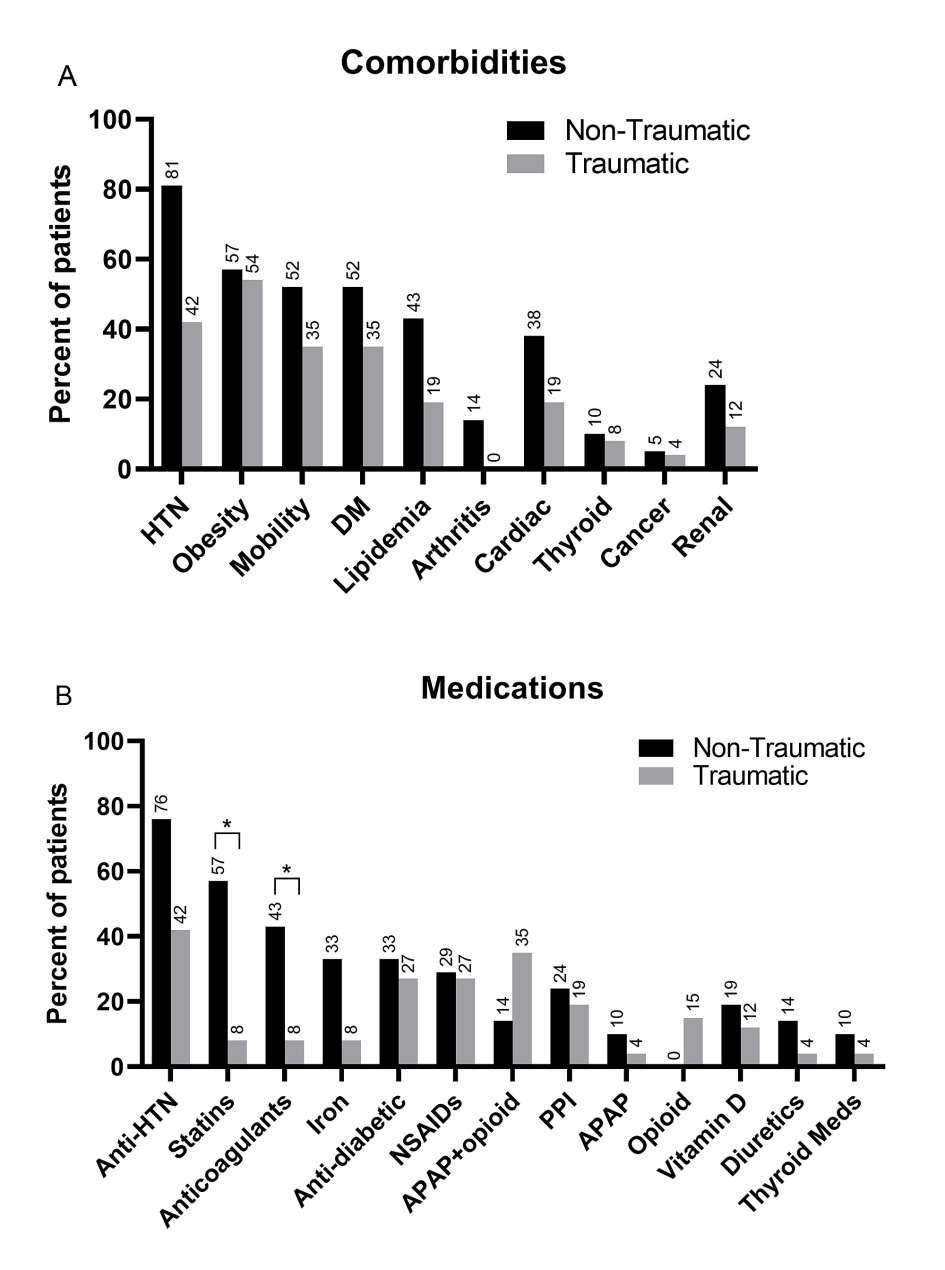
Comorbidities (A) and medications (B) reported in patients with compartment syndrome at the time of presentation. Statistical comparisons corrected for age revealed no significant differences in comorbidities between traumatic and non-traumatic groups, but statistical differences in the number of patients taking statins and anticoagulants. *p < 0.05. HTN, hypertension; DM, diabetes mellitus; PPI, proton pump inhibitors; APAP, acetaminophen; NSAIDs, non-steroidal anti-inflammatory drugs

To understand how chronic diseases and medications may affect ACS development, we quantified the top 10 comorbidities in the patients (Figure 1A) and all medications reported during the hospital stay (Figure 1B). Since the non-traumatic ACS group presented an average age older than 60, many comorbidities were expected compared to the traumatic ACS group. Statistical comparisons controlling for age revealed no significant differences in the total number of comorbidities between groups (X2 = 3.50, d.f. = 11, p > 0.05). While hypertension was twice more likely in the non-traumatic than the traumatic ACS group (Figure 1A), this difference was not significant when age was entered as a variable in the analysis.

The medications taken by the patients followed a pattern that was similar to the chronic diseases reported. The total number of medications taken by patients was not different between non-traumatic and traumatic groups when controlling for age differences (X2 = 1.03, d.f. = 1, p > 0.05). When individual classes of medications were compared between groups, the patients in the non-traumatic group had a significantly higher percentage of statins and anticoagulants use than the traumatic group, even after controlling for age differences (statins: X2 = 9.48, d.f. = 1, p < 0.05; anticoagulants: X2 = 5.23, d.f. = 1, p < 0.05). Based on this observation, we calculated the odds for using statins in the non-traumatic ACS group at 121.81 (95% CI: 3.22-4671.90), and the use of anticoagulants at 15.54 (95% CI: 1.04-231.05) compared to traumatic ACS patients. 

## Discussion

The identification and accurate diagnosis of ACS remain a challenge for healthcare providers. Here, we described the natural history of traumatic and non-traumatic compartment syndrome presentation in a cohort composed predominantly of Hispanic patients. While patients’ clinical characteristics with traumatic ACS were comparable to previous reports in the literature, the presentation of patients with non-traumatic ACS was associated with patients’ use of statins and anticoagulants compared to traumatic ACS. Various case reports on the use of satins and compartment syndrome have been published [12,13], but to our knowledge, we are the first to present a cohort of patients with this clinical presentation.

ACS presentation in traumatic injuries has been well characterized, but it is not necessarily valid for non-traumatic ACS. Some of the risk factors for non-traumatic ACS include exertional exercises, vascular surgery, blood clots, and prolonged compression of limbs [14]. Regardless of the mechanisms, both traumatic and non-traumatic presentations of ACS may lead to multiple systems involvement, including muscular, neurologic, and renal [15]. In the worst-case scenario, compartment syndrome may result in limb amputation. In cases with better outcomes, the morbidity may result in organ damage, renal failure, neuropathic pain, ulcerations, infections, etc. [16]. A conglomerate of resulting morbidities with chronic consequences may lead to a faster decline of health and increase healthcare costs. Age, pre-existing conditions, and the patient's health reserve are factors to consider during recovery. A healthy and younger individual might recover faster and reach pre-injury functional outcome levels quicker. Still, an older person with numerous comorbidities will have more difficulties getting to previous disease status. The creatinine increase in traumatic patients observed herein could be partially due to the presence of other injuries that might have produced muscle damage, dehydration, or other co-occurring clinical events that were not explored in this project. Conversely, the elevated creatinine level seen in the non-traumatic cohort could be explained by the number of comorbidities accompanying the patient before they developed ACS, mainly diabetes and hypertension. While we did not compare the patient’s baseline creatinine prior to the ACS event in this study, future studies could possibly compare how the ACS impacted baseline kidney status in traumatic vs. non-traumatic ACS events, adding to the argument about the importance of recognizing risk factors to avoid further morbidities.

The risk factors for the development of ACS include being male and younger age [17]. Thus, it is not coincidental that these risk factors overlap with the population at higher risk for traumatic injuries [18]. However, atypical presentations of ACS are equally important to recognize in all age groups. Patients of advanced age with an increased number of comorbidities, such as hypertension, have been reported to develop ACS in the absence of fractures [19]. Males were also overrepresented in the cohort of patients that develop ACS in the absence of fractures, which is somewhat different from the current observations (57% males for non-traumatic ACS). A possible explanation for this difference could arise from the female population's concomitant use of statins and anticoagulants. While 60% of females with non-traumatic ACS who used statins also used anticoagulants, only 12% of males fit the same criteria. Both anticoagulants and statins have been independently associated with increased risk of ACS development [12,13,20,21], leading us to hypothesize a synergistic effect of these drugs. Evidence for rhabdomyolysis was not documented in the medical record of the patients taking anticoagulants and statins. Still, these observations highlight the need for additional research on the contribution of pharmacological agents in ACS.

Not all statins are the same, and some may produce an increase in the risk of myopathies. Simvastatin and pitavastatin are variants of statins that become troublesome for some individuals with a defective SLC01B1 gene that encodes for an organic anion-transport polypeptide involved in the hepatocyte and myocyte uptake [22]. Hydrophilic statins are actively taken into the cells by active transporters such as those encoded by the SLC01B1 gene. In contrast, lipophilic statins are passively diffused into the liver for the CYP450 metabolism and cause fewer myopathies [23]. Some of the statins' hydrophilic nature allows for an increased uptake to the cells and therefore increased concentrations, predisposing muscles for myopathies and possibly contributing to ACS. Consequently, it may be of clinical interest to identify which type of statin a patient is taking to classify the patients at risk. Within our cohort, rosuvastatin (hydrophilic) was being used by 40% of the non-traumatic ACS patients, supporting the possible role of hydrophilic statins in myopathies that may lead to ACS. 

The contribution of race/ethnicity in the development of ACS has been largely understudied. One prior study in tibial fracture outcomes revealed an increased risk for ACS in white patients [24]. However, another study looking at post-surgical complications following lower-extremity trauma found an increased incidence of complications, including compartment syndrome, for African Americans [25]. Unfortunately, population-based reports on the incidence and outcomes of ACS in Hispanics are non-existent, with Hispanic ethnicity being described in case reports only (e.g., Reuss et al., 1999) [26]. A recent report of a young male who spontaneously developed compartment syndrome in all four limbs describes a genetic mutation in the exon 3 of the GYG1 gene, involved in glycogen storage myopathies [27], providing a novel approach for establishing a genetic predisposition to ACS. While the reasons for the lack of research studies on race/ethnicity are likely multifactorial, some of the contributing factors may be associated with the low frequency of ACS and the lack of proper identification. The increased morbidity burden associated with ACS, along with a decreased quality of life, necessitates a better understanding of how it afflicts diverse ethnic communities across the United States and abroad. 

Limitations

All retrospective studies have intrinsic limitations due to their design; nevertheless, they provide a framework for the proposal of future interventional studies. Aware of these limitations, we took advantage of the higher number of Hispanic/Latino patients living in the south Texas (USA) region to start filling the gap in Hispanics’ surgical research. The generalizability of the current data to other ethnic groups is limited due to its original design and intent. Also, Hispanic/Latino communities in other regions of the country (e.g., Los Angeles, New York, Arizona) may have different clinical characteristics than those presented here due to environmental and socioeconomic factors not considered in this study. These limitations highlight the need for studying ACS within ethnic groups across the nation using similar designs. Increasing the sample size, heterogeneity of the injuries, and pre-existing conditions will help us design predictive tools for earlier identification and treatment of ACS. The low incidence of ACS has also led to an abundance of case studies in the literature and a limited number of cohort or case-control studies. The number of interventional trials registered in the United States is relatively small (11 studies currently recruiting or active) and only two predictive or diagnosis natures. Therefore, the need for additional research on ACS is imperative. 

## Conclusions

While clinical guidelines for ACS diagnosis have been published, the current tests, including biomarkers and imaging modalities, still show low sensitivity and specificity. A list of risk factors for ACS development after either traumatic or non-traumatic events have been reported, but how comorbidities might influence ACS's outcome is not well understood. Risk factor analyses for ACS should be improved to include comorbidities and medications within an easy-to-use bed-side scoring system. Based on the presented data, patients taking anticoagulants and statins should be carefully monitored for ACS development, especially after non-traumatic qualifying events and advanced age. Further studies should be aimed at identifying how statins interact with the patient's genetic profile and other comorbidities (e.g., obesity, diabetes, hyperlipidemia) to increase the risk of ACS.

## References

[1] Via AG, Oliva F, Spoliti M, Maffulli N (2015). Acute compartment syndrome. Muscles Ligaments Tendons J.

[2] Shadgan B, Menon M, Sanders D (2010). Current thinking about acute compartment syndrome of the lower extremity. Can J Surg.

[3] Garner MR, Taylor SA, Gausden E, Lyden JP (2014). Compartment syndrome: diagnosis, management, and unique concerns in the twenty-first century. HSS J.

[4] Vaillancourt C, Shrier I, Vandal A, Falk M, Rossignol M, Vernec A, Somogyi D (2004). Acute compartment syndrome: how long before muscle necrosis occurs?. Can J Emerg Med.

[5] United States Census Bureau: United States Census Bureau: Census Data Mapper (2020). United States Census Bureau: Census Data Mapper. https://datamapper.geo.census.gov/map.html.

[6] (2020). Centers for Disease Control and Prevention: National diabetes statistics report. https://www.cdc.gov/diabetes/pdfs/data/statistics/national-diabetes-statistics-report.pdf.

[7] (2020). American Diabetes Association: The burden of diabetes in Texas. http://main.diabetes.org/dorg/PDFs/Advocacy/burden-of-diabetes/texas.pdf.

[8] University of Texas Border Health Office: University of Texas RGV Border Health Office Diabetes Registry (2020). University of Texas Border Health Office: University of Texas RGV Border Health Office Diabetes Registry. https://www.utrgv.edu/bho/diabetes-registry/statistics/index.htm.

[9] Ramirez AG, Thompson IM, Vela L (2013, pp. 1-138). The South Texas Health Status Review: A Health Disparities Roadmap. South Texas Heal Status Rev A Heal Disparities Roadmap.

[10] Rasul AT (2020). Acute compartment syndrome. Medscape/Drugs Dis Phys Med Rehabil.

[11] Modrall JG (2019). Compartment syndrome and its management. Rutherford’s Vascular and Endovascular Surgery.

[12] Flamini S, Zoccali C, Persi E, Calvisi V (2008). Spontaneous compartment syndrome in a patient with diabetes and statin administration: a case report. J Orthop Traumatol.

[13] Webber MA, Mahmud W, Lightfoot JD, Shekhar A (2004). Rhabdomyolysis and compartment syndrome with coadministration of risperidone and simvastatin. J Psychopharmacol.

[14] (2020). American Academy of Orthopaedic Surgeons: Compartment Syndrome. OrthoInfo. https://orthoinfo.aaos.org/en/diseases--conditions/compartment-syndrome/.

[15] Lundy DW, Bruggers JL (2019). Management of Missed Compartment Syndrome. Compartment Syndrome.

[16] Igoumenou VG, Kokkalis ZT, Mavrogenis AF (2019). Fasciotomy Wound Management. Compartment Syndrome.

[17] McQueen MM, Gaston P, Court-Brown CM (2000). Acute compartment syndrome. Who is at risk?. J Bone Joint Surg Br.

[18] (2020). Centers for Disease Control and Prevention. WISQARS: Leading Causes of Death Reports, 1981 - 2018. https://webappa.cdc.gov/sasweb/ncipc/leadcause.html.

[19] Hope MJ, McQueen MM (2004). Acute compartment syndrome in the absence of fracture. J Orthop Trauma.

[20] Zimmerman DC, Kapoor T, Elfond M, Scott P (2013). Spontaneous compartment syndrome of the upper arm in a patient receiving anticoagulation therapy. J Emerg Med.

[21] Richards AM, Moss AL (1997). Biceps rupture in a patient on long-term anticoagulation leading to compartment syndrome and nerve palsies. J Hand Surg Br.

[22] Moßhammer D, Schaeffeler E, Schwab M, Mörike K (2014). Mechanisms and assessment of statin-related muscular adverse effects. Br J Clin Pharmacol.

[23] Schachter M (2005). Chemical, pharmacokinetic and pharmacodynamic properties of statins: an update. Fundam Clin Pharmacol.

[24] Piposar J, Fowler JR, Gaughan JP, Rehman S (2012). Race may not affect [correct] outcomes in operatively treated tibia fractures. Clin Orthop Relat Res.

[25] Low EE, Inkellis E, Morshed S (2017). Complications and revision amputation following trauma-related lower limb loss. Injury.

[26] Reuss PM, Rosen RJ, Adelman M (1999). Compartment syndrome complicating lower extremity thrombolysis. J Vasc Interv Radiol.

[27] Joseph VM, Nagy MT, Akhtar S, Ng CY (2020). Sequential spontaneous compartment syndrome in multiple limbs in a young adult with GYG1 gene mutation. BMJ Case Rep.

